# Biochemical and Genomic Characterization of Two New Strains of *Lacticaseibacillus paracasei* Isolated from the Traditional Corn-Based Beverage of South Africa, Mahewu, and Their Comparison with Strains Isolated from Kefir Grains

**DOI:** 10.3390/foods12010223

**Published:** 2023-01-03

**Authors:** Konstantin V. Moiseenko, Anna V. Begunova, Olga S. Savinova, Olga A. Glazunova, Irina V. Rozhkova, Tatyana V. Fedorova

**Affiliations:** 1 A. N. Bach Institute of Biochemistry, Research Center of Biotechnology, Russian Academy of Sciences, Leninsky Ave. 33/2, 119071 Moscow, Russia; 2 Federal State Budgetary Scientific Institution “All-Russian Research Institute of Dairy Industry”, 115093 Moscow, Russia

**Keywords:** *Lacticaseibacillus paracasei*, mahewu, kefir grains, genome sequencing, antibacterial activity, milk fermentation, proteolytic activity, antioxidant activity, angiotensin-converting enzyme inhibitory activity (ACE-I)

## Abstract

*Lacticaseibacillus paracasei* (formerly *Lactobacillus paracasei*) is a nomadic lactic acid bacterium (LAB) that inhabits a wide variety of ecological niches, from fermented foodstuffs to host-associated microenvironments. Many of the isolated *L. paracasei* strains have been used as single-strain probiotics or as part of a symbiotic consortium within formulations. The present study contributes to the exploration of different strains of *L. paracasei* derived from non-conventional isolation sources—the South African traditional fermented drink mahewu (strains MA2 and MA3) and kefir grains (strains KF1 and ABK). The performed microbiological, biochemical and genomic comparative analyses of the studied strains demonstrated correlation between properties of the strains and their isolation source, which suggests the presence of at least partial strain adaptation to the isolation environments. Additionally, for the studied strains, antagonistic activities against common pathogens and against each other were observed, and the ability to release bioactive peptides with antioxidant and angiotensin I-converting enzyme inhibitory (ACE-I) properties during milk fermentation was investigated. The obtained results may be useful for a deeper understanding of the nomadic lifestyle of *L. paracasei* and for the development of new starter cultures and probiotic preparations based on this LAB in the future.

## 1. Introduction

Lactic acid bacteria (LAB) are a heterogeneous group of microorganisms that play a key role in various food fermentation processes [1,2]. In addition to advantageous storage and organoleptic properties, many foods fermented by LAB possess additional health-promoting benefits, such as imparting improvements to digestion and tolerance to lactose [3], hypocholesterolemic and antihypertensive effects [4,5] and antioxidant and anticarcinogenic activities [6,7]. Additionally, LAB itself can possess probiotic properties, such as the ability to impart improvements to the intestinal barrier and commensal microbial balance [8,9], production of beneficial enzymes (e.g., β-galactosidase and bile salt hydrolase) and neurochemicals [10,11], suppression of pathogenic microflora [12], and modulation of the immune system [13].

Among the LAB, *Lactobacillus* is the most well-known genus and currently comprises more than 200 species with extremely diverse phenotypes, genotypes and ecology [14]. Moreover, for each actively studied species of *Lactobacillus*, more than 100 strains are currently described and have genomes sequenced [15]. Although several researchers have proposed the idea that some beneficial properties of *Lactobacillus* spp. may be innate attributes of taxonomic ranks higher than the strain [16], the strain-specificity of such properties is still a cornerstone principle of probiotic science. Therefore, the isolation of individual strains of *Lactobacillus* spp. and the exploration of their beneficial properties are necessary, albeit tedious, undertakings to develop new associations of starter cultures for products with pronounced health benefits [17,18,19].

The present study contributes to the exploration of different strains of *Lacticaseibacillus paracasei* (formerly *Lactobacillus paracasei*). Many of the isolated *L. paracasei* strains have been used as single-strain probiotics or as part of a symbiotic consortium within formulations [20]. Examples actively marketed around the name “probiotic strains of *L. paracasei*” are: *L. paracasei* F19 from Chr. Hansen, which is mainly used as a part of the starter culture in the popular Scandinavian yogurt from the Arla Foods company; *L*. *paracasei* DG (*L*. *paracasei* CNCM I-1572), which is mainly used as a single-strain probiotic in the Enterolactis food supplement from the Italian company SOFAR; and *L*. *paracasei* Shirota, which is mainly used for the preparation of the Japanese sweetened probiotic milk beverage Yakult prom the Yakult Honsha company.

*L. paracasei* is closely related to such widely researched and used probiotic species as *Lacticaseibacillus casei* and *Lacticaseibacillus rhamnosus* and forms with these species a distinctive evolutionary/taxonomic group—the *L. casei* group [21,22]. The species of the *L. casei* group populate a wide variety of different niches, from fermented foodstuffs to host-associated microenvironments, and represent a paradigmatic “nomadic species” [23,24]. Although nomadic species are not permanent residents of intestinal ecosystems, they may persist in them for at least a limited time [23]. Additionally, when isolated from environments other than host-associated (e.g., commercial or artisanal dairy products and plants), the isolation sources of nomadic species are not well-correlated with the evolution of their genomes [25]. Consequently, no niche-specific adaptations common to the majority of strains isolated from the same source can be identified [26]. However, it is easily possible for the different strains of nomadic species to possess different independent adaptations to the same environment [24,27]. For example, Smocvina et al. [26] reported that dairy-derived strains of *L. paracasei* generally possessed reduced genome size with a smaller number of sugar cassettes. The inherent genetic flexibility of nomadic strains has made these strains a natural library of evolutionarily selected variations yet to be employed for biotechnological applications [28].

Currently, 310 strains of *L. paracasei* with sequenced genomes have been reported [15]; however, there are several pieces of evidence suggesting that not all the strain biodiversity of this species has been explored. Firstly, *L. paracasei* is considered to have an open pan-genome [29]; hence, the number of new gene families increases with the addition of the genomes from new strains [30]. Secondly, there is a limited number of strains isolated from non-conventional sources, examples of which would be non-dairy fermented foodstuff and symbiotic cultures of bacteria and yeast (SCOBY), such as kefir grains and kombucha [25]. The GeneBank database contains only eight genomes of *L. paracasei* isolated from kefir or kefir grains and only one from non-dairy fermented foodstuff (beer).

In this article, we report a comparative biochemical and genomic characterization of four strains of *L. paracasei* isolated from non-conventional sources: the strains *L. paracasei* MA2 and *L. paracasei* MA3 were isolated from the traditional, corn-based, nonalcoholic beverage of South Africa mahewu in the course of this work; and the strains *L. paracasei* KF1 and *L. paracasei* ABK were previously isolated from the SCOBY traditionally used in Commonwealth of Independent States (CIS) countries for preparation of kefir and kefir grains [31]. Additionally, for the studied strains, antagonistic activities against common pathogens and against each other were observed, and the ability to release bioactive peptides with antioxidant and angiotensin I-converting enzyme inhibitory (ACE-I) properties during milk fermentation was investigated.

## 2. Materials and Methods

### 2.1. Isolation, Identification and Profile of Enzymatic Activities

The strains *L. paracasei* MA2 and *L. paracasei* MA3 were isolated from mahewu, the samples of which were purchased in the distribution network of Durban (South Africa) and analytically characterized by Moiseenko et al. [32]. The isolation procedure was performed as described by Begunova et al. [31]. In brief, a series of tenfold dilutions were inoculated into MRS (De Man, Rogosa and Sharpe) broth with the addition of 10% ethanol and cultivated at 30 ± 1 °C for 3–5 days to enrich the medium with lactobacilli. An enriched culture of lactobacilli was inoculated on MRS agar (pH 5.4) and anaerobically incubated at a temperature of 30 ± 1 °C for 3–5 days. Morphologically identical colonies were selected for further cultivation in MRS broth.

The isolated lactobacilli were biochemically characterized using API 50CH (BioMerieux, Marcy l’Etoile, France) and API ZYM (BioMerieux) test systems according to the manufacturer’s instructions. The results of the API 50CH test were analyzed with the APIWEB web server (https://apiweb.biomerieux.com, accessed on 24 September 2022). The genotyping of the obtained isolates was performed with colony polymerase chain reaction (PCR) from the MRS agar plates. The 16S rRNA gene was amplified according to [33] with a Taq DNA polymerase kit (Evrogen, Moscow, Russia) and the primers bak11w (5’-AGT TTG ATC MTG GCT CAG-3’) and bak4 (5’-AGG AGG TGA TCC ARC CGC A-3’). Successfully amplified PCR products were extracted from 2% agarose gel with a commercial QIAquick Gel Extraction Kit (Qiagen, Valencia, CA, USA). Sanger sequencing of the purified PCR products was carried out with the same primers as for PCR. The construction of the phylogenetic tree with obtained 16S rRNA sequences, as well as sequences from various *Lactobacillus* species type strains [14], was performed using the RAxML-HPC BlackBox (v 8.2.10) program [34] at the CIPRES Science Gateway [35].

The strains *L. paracasei* KF1 and *L. paracasei* ABK were obtained from the Microorganism Collection of the All-Russia Research Institute of the Dairy Industry (VNIMI, Moscow, Russia). Both strains were isolated from kefir grains and biochemically characterized using API 50CH and API ZYM test systems by Begunova et al. [31]. The sequences of the 16S ribosomal RNA genes of these strains can be found at the GenBank accessory numbers MW558119.1 and MN994625.1 for *L. paracasei* KF1 and *L. paracasei* ABK, respectively.

### 2.2. Inhibition of Pathogens and Antagonistic Interactions

The ability of the *L. paracasei* KF1, *L. paracasei* ABK, *L. paracasei* MA2 and *L. paracasei* MA3 to inhibit common pathogens was assessed according to Begunova et al. [36]. The pathogenic bacterium strains *Staphylococcus aureus* ATCC-6538 and *Escherichia coli* ATCC-25922 were purchased from the American Type Culture Collection (Manassas, VA, USA), and *Salmonella typhimurium* NCTC 00074 was purchased from the National Collection of Type Cultures (Salisbury, UK). In brief, the antagonistic activity was evaluated with the co-culture method. For the experimental samples, 20 mL of MRS broth was simultaneously inoculated with 1 mL (approximately 10^7^ CFU·mL^−1^) of the studied *L. paracasei* strain and 1 mL (approximately 10^7^ CFU·mL^−1^) of the pathogenic test-strain. The single-species cultivations of the pathogenic bacteria were used as a control. The incubation was carried out at 37 ± 2 °C, and samples were collected after 24 and 48 h. Pathogen cells were counted on commercial mediums based on pancreatic sprat hydrolysate, SPA agar medium (Mikrogen, Moscow, Russia), at 37 °C for 24–48 h.

The antagonistic interactions between the studied *L. paracasei* strains were assessed with the perpendicular streak test following Savinova et al. [37]. At the first stage, the pre-culture of the first LAB was streaked on the MRS agar and incubated under anaerobic conditions at 37 °C for 24 h. At the second stage, the pre-culture of the second LAB was streaked perpendicularly to the first LAB, and the plate was incubated under anaerobic conditions for another 24 h at 37 °C. The antagonistic interactions between LAB were assessed visually from the presence of a growth inhibition zone.

### 2.3. Fermentation of Milk

Growth characteristics and functional properties of the *L. paracasei* KF1, *L. paracasei* ABK, *L. paracasei* MA2 and *L. paracasei* MA3 were studied during fermentation of the skim milk. Sterile skim milk was inoculated with 1% of the corresponding strain and incubated at 30 °C for 72 h. Samples were collected under sterile conditions at 6, 16, 24, 48 and 72 h of fermentation, and the number of viable cells (colony-forming units (CFUs)) was counted on MRS agar and the pH was measured using a Seven Easy pH meter (Mettler Toledo, Greifensee, Switzerland).

For the measurements of proteolytic activity, antioxidant-capacity and ACE-I-activity protein-peptide fractions were isolated from fermented milk. For the samples with a pH above 4.6, the pH was adjusted to 4.6 by adding 0.75% trichloroacetic acid (TCA). The samples were centrifuged for 30 min at 4 °C and 10,000× *g* in a 5702R centrifuge (Eppendorf, Germany). The obtained supernatants were filtered through 0.45 μm PVDF syringe filters (Sartorius, Germany). The obtained protein-peptide fractions were frozen and stored at −80 °C until further analysis. Before the analysis, samples were thawed and additionally filtered with 0.45 μm polyvinylidene difluoride (PVDF) syringe filters.

The proteolytic activity was determined quantitatively as the amount of released amino groups using the 2,4,6-trinitrobenzenesulfonic acid (TNBS) method [38], as described by Torkova et al. [39]. The results were expressed as the amount of L-Leu molar equivalents, mM(L-Leu).

The antioxidant capacity of the samples was determined with the oxygen radical absorbance capacity fluorescence method (ORAC) with the generation of peroxyl radicals in the reaction medium, as described by Torkova et al. [39]. The results were expressed as the amount of Trolox molar equivalents, µM(TE).

The in vitro hypotensive effect of the fermented milks was assessed as the angiotensin-I-converting enzyme (ACE)-inhibiting activity of the samples (ACE-I activity), as described by Torkova et al. [39]. The measurements were performed with a BioTek Synergy 2 microplate photometer–fluorometer (BioTek). The results were expressed as the half maximal inhibitory concentration IC_50_ (reported as mg of protein per mL).

### 2.4. Genome Sequencing, Assembly and Annotation

The DNA isolation, genome sequencing and genome annotation of *L. paracasei* KF1, *L. paracasei* ABK, *L. paracasei* MA2 and *L. paracasei* MA3 were performed as described by Savinova et al. [37]. In brief, total DNA was extracted from liquid MRS cultures using a DNeasy mericon Food Kit (Qiagen) according to the manufacturer’s protocol. The DNA library was prepared using the Ion AmpliSeq library kit 2.0 (Thermo Fisher Scientific, Waltham, MA, USA) and indexed with an Ion Xpress barcode adapters 1–16 kit (Thermo Fisher Scientific, MA, USA). Whole-genome sequencing was carried out using the Ion Torrent Personal Genome Machine (PGM) (Thermo Fisher Scientific). The obtained reads were pre-processed and assembled with CLC Genomics Workbench 11.0 (Qiagen). Upon submission, genome annotations were performed using NCBI Prokaryotic Genome Annotation Pipeline (PGAP) [40]. Additionally, annotation with ISfinder [41], PHASTER [42], PlasmidFinder [43] and BAGEL4 [44] was performed on the web. The comparative genome analysis was performed using the Anvi’o suite of programs [45,46].

### 2.5. Statistical Data Manipulations

All experiments were performed in three biological replicates. All statistical comparisons were firstly performed using a one-way ANOVA omnibus *F*-test. When a significant (*p* < 0.05) value for the *F*-statistics was found, differences between means were evaluated using Tukey’s honestly significant difference (HSD) multiple comparison test (*p* < 0.05).

## 3. Results and Discussion

### 3.1. Isolation and Taxonomic Assignment of the Mahewu-Derived Strains

**Figure 1:**
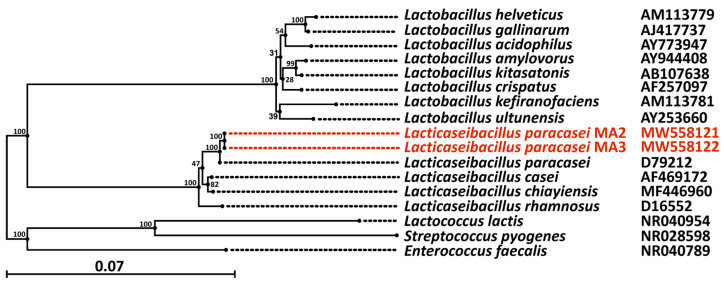
Phylogenetic tree constructed using 16S rRNA sequences of various *Lactobacillus* species type strains [14] and 16S rRNA sequences of strains isolated from mahewu in this work (marked in red). The evolutionary distance corresponding to one change every 100 nucleotides is shown by the scale. The number over the nodes corresponds to their bootstrap values.

As a result of a series of successive subcultures on the media selective for lactobacilli, several rod-shaped (approximately 1 µkm in length) Gram-positive bacteria were isolated. The isolated bacteria formed small, round, creamy-yellow non-transparent colonies (approximately 1 mm in diameter) with smooth edges on MRS agar. To identify the isolated strains, their 16S rRNA genes were sequenced and compared with the sequences of the type strains registered in the GenBank database. As a result, two isolated strains demonstrated 99.9% similarity with the type strains of *L. paracasei*. These strains were named *L. paracasei* MA2 and *L. paracasei* MA3 and were deposited into the Microorganism Collection of the All-Russia Research Institute of the Dairy Industry (VNIMI, Moscow, Russia). The obtained sequences of 16S rRNA were deposited into GenBank under the accession numbers MW558121.1 and MW558122.1 for *L. paracasei* MA2 and *L. paracasei* MA3, respectively. The phylogenetic tree constructed using 16s rRNA sequences of various *Lactobacillus* species type strains [14] and 16S rRNA sequences of the mahewu-derived strains *L. paracasei* MA2 and *L. paracasei* MA3 is shown in Figure 1.

### 3.2. Comparative Functional Characterization of the Mahewu- and Kefir-Derived Strains

#### 3.2.1. Biochemical Characterization: Ability to Utilize Different Substrates and Profile of Enzymatic Activities

**Figure 2:**
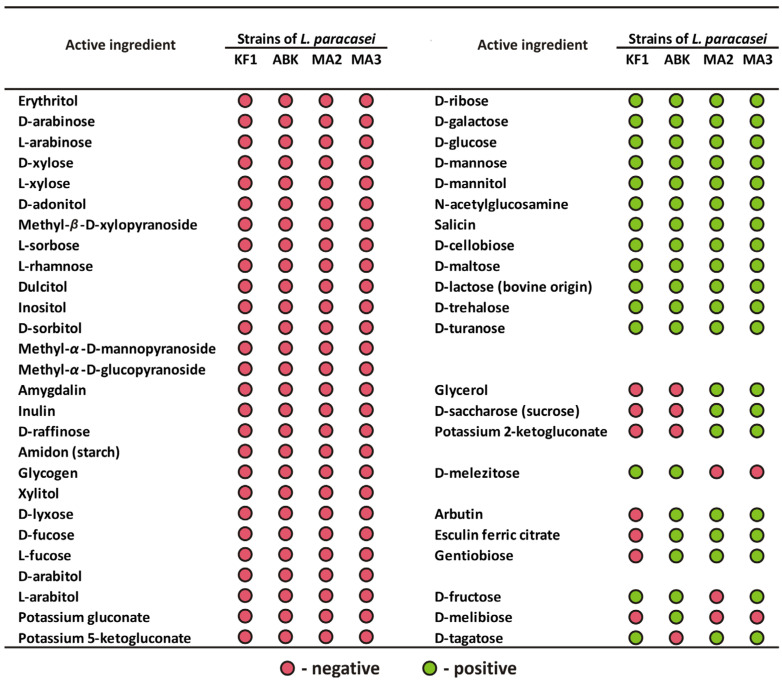
The API 50 substrate utilization profile of the kefir-derived (KF1 and ABK) and mahewu-derived (MA1 and MA2) strains of *L. paracasei*. The data for the kefir-derived strains were obtained from Begunova et al. [31].

The ability of the mahewu-derived strains to utilize 49 different substrates was assessed using an API 50 test system. The obtained substrate-utilization patterns for both *L. paracasei* MA2 and *L. paracasei* MA3 were 91% identical to that typical for *L. paracasei* species, according to the APIWEB database. The comparison of the API 50 profile of the mahewu-derived strains with that of the kefir-derived strains, previously described by Begunova et al. [31], is presented on Figure 2.

As was expected for different strains of the same species, all four studied strains of *L. paracasei* were similar in their use of 39 out of 49 (i.e., 79.6%) tested substrates, 12 of which were used by all strains and 27 not by any strain. Notably, for four substrates (i.e., 8.2% of the total), the usage pattern clearly separated strains isolated from kefir and mahewu: only mahewu-derived strains were able to utilize glycerol, D-saccharose (sucrose) and potassium 2-ketogluconate, while D-melezitose was utilized only by kefir-derived strains. Three substrates (i.e., 6.1% of the total), arbutin, esculin ferric citrate and gentiobiose, were utilized by all strains except *L. paracasei* KF1. In addition, for three substrates (i.e., 6.1% of the total), strain-specific features of utilization were observed: only *L. paracasei* ABK demonstrated the ability to utilize D-melibiose and the inability to utilize D-tagatose, and only *L. paracasei* MA2 was not able to utilize D-fructose.

**Figure 3:**
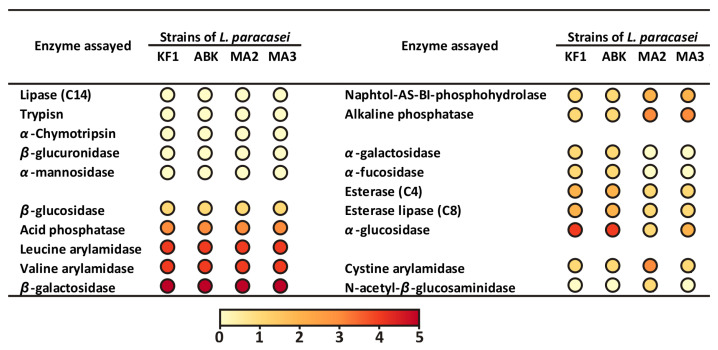
The API ZYM enzymatic activity profiles of kefir-derived (KF1 and ABK) and mahewu-derived (MA2 and MA3) strains of *L. paracasei*. The data for the kefir-derived strains were obtained from Begunova et al. [31].

The enzymatic activities of the mahewu-derived strains were assessed with a semi-quantitative API ZYM test system, which detects 19 enzymatic activities, including those of glycoside-hydrolases, proteases, phosphatases, and esterases. The comparison of the API ZYM profile of the mahewu-derived strains with that of the kefir-derived strains, previously described by Begunova et al. [31], is presented on Figure 3.

All four studied *L. paracasei* strains demonstrated similar results for the majority of the tested enzymatic activities (10 out of 19 or 52.6%): five activities were absent for all strains, and five activities were detected at the same semi-quantitative level for all strains. For seven enzymatic activities (i.e., 36.8% of the total), the activity pattern clearly separated strains isolated from kefir and mahewu: while the mahewu-derived strains demonstrated higher activities of naphtol-AS-BI-phosphohydrolase and alkaline phosphatase, the activities of *α*-galactosidase, *α*-fucosidase, esterase (C4), esterase lipase (C8) and *α*-glucosidase were higher for kefir-derived strains. The *L. paracasei* MA2 demonstrated the highest cystine arylamidase activity among other strains and was the only strain with detectable N-acetyl-β-glucosaminidase activity.

It is worth comparing the results of the API 50 test with the results of the API ZYM test in terms of contrasting biochemical properties of strains derived from kefir and mahewu. In the API 50 tests, only 8.2% of all tested substrates demonstrated clear separation of these two groups of strains. Although this figure is small, it is arguably still larger than can be expected from random chance. Hence, based on the API 50 data, it was possible to hypothesize that specific differences between strains can be correlated with strains’ isolation source. The data on the API ZYM test clearly substantiated this hypothesis, since 36.8% of all tested activities demonstrated the clear boundary between kefir- and mahewu-derived strains.

#### 3.2.2. Inhibition of Pathogens and Antagonistic Interactions

**Figure 4:**
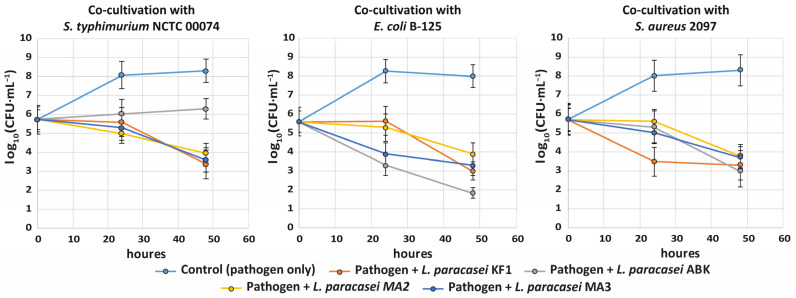
Changes in the viable cell count of *S. typhimurium* NCTC 00074, *E. coli* B-125 and *S. aureus* 2097 during single- and two-species cultivations with the kefir-derived (KF1 and ABK) and mahewu-derived (MA2 and MA3) strains of *L. paracasei*. The error bars represent the standard deviations from the mean.

To examine antagonistic activity of the studied *L. paracasei* strains against planktonic cells of the pathogenic bacteria, two-species co-cultivations were performed. The following strains of pathogenic bacteria were used: *S. typhimurium* NCTC 00074, *S. aureus* 2097 and *E. coli* B-125. The single-species cultivations of the pathogenic bacteria were used as a control. The dynamics of changes in the viable cell count of the pathogenic bacteria are shown in the Figure 4.

While intensive growth of pathogenic bacterial strains was observed in their monoculture (control), in co-cultivations with the studied *L. paracasei* strains, their viable cell count constantly decreased. Generally, all the studied *L. paracasei* strains demonstrated similar abilities for inhibition of pathogens. The most prominent decrease, by three to four orders of magnitude in 48 h, was observed for *E. coli* B-125. The viable cell counts of other pathogens decreased by approximately two orders of magnitude. The most distinctive strain was *L. paracasei* ABK. Compared to other strains, this strain demonstrated the best inhibition for *E. coli* B-125, and the worst for *S. typhimurium* NCTC 00074.

The antagonistic interactions between studied strains of *L. paracasei* were tested during their solid-state co-cultivation with the perpendicular streak method (Table 1). Although strains did not demonstrate pronounced antagonism to each other, some weak antagonistic interactions were detected. Interestingly, while no antagonistic interactions were detected between the kefir-derived strains or between the mahewu-derived strains, the mahewu-derived strains were able to weakly suppress the growth of the kefir-derived strains.

#### 3.2.3. Growth Ability, Acidification Capability and Proteolytic Activity during Milk Fermentation

**Figure 5:**
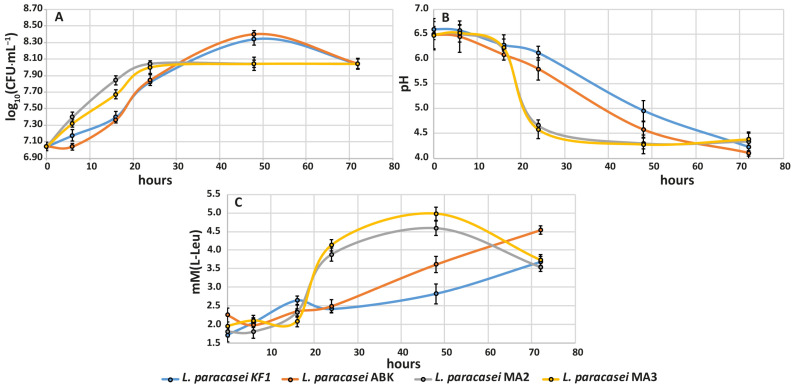
Fermentation characteristics of the kefir-derived (KF1 and ABK) and mahewu-derived (MA2 and MA3) strains of *L. paracasei* during milk fermentation: (**A**) the dynamics of change in the viable cell count; (**B**) the dynamics of change in the pH value; (**C**) the dynamics of change in the proteolytic activity. The error bars represent the standard deviations from the mean.

The dynamics of changes in the viable cell count are shown in Figure 5A. During the first 24 h of the fermentation process, the kefir-derived strains demonstrated lower growth rates compared to the mahewu-derived strains. After 24 h of fermentation, the kefir-derived strains continued to grow until 48 h, when they achieved the maximum viable cell count of (2.35 ± 1.07) × 10^8^ CFU·mL^−1^, and then their viable cell count decreased to (1.10 ± 1.06) × 10^8^ CFU·mL^−1^ at 72 h of fermentation. In contrast, the mahewu-derived strains stopped their growth after 24 h of fermentation, when they achieved the maximum viable cell count of (1.10 ± 0.91) × 10^8^ CFU·mL^−1^, and then their viable cell count remained constant until 72 h of fermentation.

The dynamics of changes in the pH values are shown in Figure 5B. As expected, the acidification capabilities of all studied strains negatively correlated with their viable cell count. The faster the strain grew, the faster the acidification of its medium was observed to be. The mahewu-derived strains showed a pH decrease of approximately two units in a 24 h timespan and reached the final pH of 4.3 ± 0.2 at 72 h of fermentation. The kefir-derived strains gradually decreased their pH by approximately 0.04 pH units per hour, reaching the same final pH value as mahewu-derived strains at the end of the fermentation. While the mahewu-derived strains were already able to form clots after 24 h of fermentation, because of casein precipitation at pH lower than 4.6 [47], the clots in the milk fermented by kefir-derived strains were observed only after 72 h of fermentation.

The dynamics of changes of the proteolytic activity for the studied strains are shown in Figure 5C. For all strains, the proteolytic activity until 16 h of fermentation was almost the same and did not differ significantly from that at the beginning of fermentation. After 16 h of fermentation, the proteolytic activity of the mahewu-derived strains rose rapidly up to 3.88 ± 0.21 mM(L-Leu) at 24 h of fermentation and continued to increase up to 4.78 ± 0.22 mM(L-Leu) at 48 h. At 72 h of fermentation, the proteolytic activity of the mahewu-derived strains decreased down to 3.63 ± 0.15 mM(L-Leu). In contrast, the proteolytic activity of the kefir-derived strains gradually increased from 16 to 72 h of fermentation by approximately 0.04 and 0.02 mM(L-Leu) per hour, reaching the final values of 4.61 ± 0.15 and 3.68 ± 0.15 mM(L-Leu) at the end of the fermentations for *L. paracasei* KF1 and *L. paracasei* ABK, respectively.

Hence, all three studied parameters—growth ability, acidification capability and proteolytic activity—generally correlated with each other and with the origin of the strains during milk fermentation. The kefir-derived strains demonstrated slower growth, acidification capability and proteolytic activity compared to the mahewu-derived strains. Although slow growth and acidification of milk by the kefir-derived strain could present hindrances for the technological use of these strains, it could be an advantageous property from the probiotic perspective. Recently, Jung et al. [48] demonstrated that, in some cases of milk fermentation by *Lacticaseibacillus casei*, the slow-growing strains improved several of their probiotic characteristics (e.g., resistance to simulated gastrointestinal digestion and intestinal adhesion ability) after long-term fermentation.

#### 3.2.4. Development of Antioxidant and Antihypertensive Properties during Milk Fermentation

**Figure 6:**
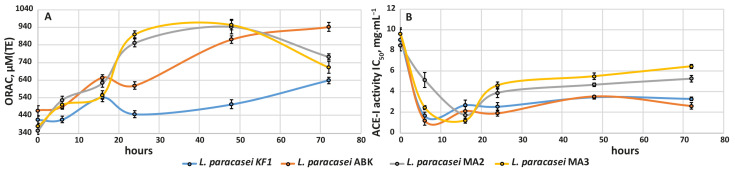
The development of antioxidant and ACE-I activities in the milk fermented by the kefir-derived (KF1 and ABK) and mahewu-derived (MA2 and MA3) strains of *L. paracasei*: (**A**) the development of antioxidant activity; (**B**) the development of ACE-I activity. The error bars represent the standard deviations from the mean.

The development of antioxidant activity during fermentation of milk by the studied strains is shown in Figure 6A. The antioxidant activity of milk fermented by the kefir-derived strains steadily increased over the entire fermentation time at approximately 2.7 and 6.9 µM(TE) per hour for *L. paracasei* KF1 *L. paracasei* ABK, respectively. For milk fermented by the mahewu-derived strains, the rapid growth of antioxidant activity up to 850 ± 23 µM(TE) was observed in the first 24 h of fermentation. From 24 to 48 h, the antioxidant activity stayed at almost the same level, after which it slightly decreased until the end of fermentation, reaching approximately 750 ± 33 µM(TE). Generally, the antioxidant activity of the fermented milk correlated with the strains’ proteolytic activity. Previously, we have discussed a similar situation for *Lactobacillus helveticus*, *Lactobacillus rhamnosus* and *Lactobacillus reuteri* [49] and hypothesized that the main reason for this correlation is the not very stringent requirements that peptides must meet in order to possess reasonable antioxidant activity [50,51,52].

The development of ACE-I activity is shown in Figure 6B. For milk fermented by all studied strains, the most active increase in ACE-I activity (i.e., decrease in the IC_50_) was observed in the first 16 h of fermentation; at this time, the IC_50_ reached almost the same value for all strains at approximately 2.0 ± 0.4 mg·mL^−1^. For milk fermented by the kefir-derived strains, the value of IC_50_ did not change until the end of the fermentation. For the mahewu-derived strains, the value of IC_50_ slightly increased at the end of fermentation, reaching approximately 5.5 ± 0.33 mg·mL^−1^. The slower decrease in ACE-I activity in milk fermented by the kefir-derived strains may not only indicate the possibility of longer storage of this milk but also, once again, underline the possibility of using these strains in long-term fermentations without loss of ACE-I properties in the fermented products.

Currently, there is only one published article describing the ACE-I activity of the milk fermented by *L. paracasei* (strain L26) authored by Donkor et al. [53], in which an IC_50_ of 0.196 ± 0.008 mg·mL^−1^ was reported. Although the IC_50_ reported in our article (2.0 mg·mL^−1^) is substantially higher, it is still in the range typical for milk fermented with *Lactobacillus* spp. [54].

### 3.3. Comparative Genomic Characterization of the Mahewu- and Kefir-Derived Strains

#### 3.3.1. Genome Sequencing, Assembly and Annotation

Using Ion Torrent technology, the draft genomes of *L. paracasei* KF1, *L. paracasei* ABK, *L. paracasei* MA2 and *L. paracasei* MA3 were sequenced with overall coverage of 100× and ultimately assembled into 248, 246, 363 and 350 contigs, respectively (Table 2). For *L. paracasei* KF1, the N50 value was 36,612 bp, with the longest contig being 212,858 bp and the mean contig size 10,572 bp. For *L. paracasei* ABK, the N50 value was 36,610 bp, with the longest contig being 212,860 bp and the mean contig size 10,773 bp. For *L. paracasei* MA2, the N50 value was 37,017 bp, with the longest contig being 170,621 bp and the mean contig size 7622 bp. For *L. paracasei* MA3, the N50 value was 37,018 bp, with the longest contig being 170,654 bp and the mean contig size 8022 bp. The final size of the assemblies was 2.7 and 2.9 Mb for the kefir-derived (KF1 and ABK) and mahewu-derived (MA2 and MA3) strains, respectively. The Whole Genome Shotgun projects were deposited at DDBJ/ENA/GenBank under the accessions GCA_023470645, GCA_018967025, GCA_018966985 and GCA_023470655 for *L. paracasei* KF1, *L. paracasei* ABK, *L. paracasei* MA2 and *L. paracasei* MA3, respectively. The versions described in this paper are versions GCA_023470645.1, GCA_018967025.1, GCA_018966985.1 and GCA_023470655.1 for *L. paracasei* KF1, *L. paracasei* ABK, *L. paracasei* MA2 and *L. paracasei* MA3, respectively. All the sequenced genomes belong to two BioProgects—PRJNA824719 and PRJNA736961. In general, the obtained assemblies and annotations of the genomes of the two kefir-derived (KF1 and ABK) and two mahewu-derived (MA2 and MA3) strains of *L. paracasei* were of comparable quality to previously published genomes of other *L. paracasei* strains [15].

For all studied strains, the genome sizes and numbers of predicted CDSs were in the previously identified ranges—2.5–4 Mb and 2200–3200 CDSs—for free-living and nomadic *Lactobacillus* spp. [23]. It should be especially emphasized that the mahewu-derived strains possessed a 200 kb larger genome size (approximately 180 additional genes) compared to the kefir-derived strains. Additionally, the genomes of the mahewu-derived strains contained 50 more pseudogenes (i.e., the genes that have been silenced by one or more deleterious mutations).

Since pseudogenes can persist in bacterial genomes over a long evolutionary period, they can usually be thought of as “archaeological records” of pre-existing but now extinct proteins, enzymes or even entire biological pathways [55,56]. The accumulation of pseudogenes in the genomes of the mahewu-derived strains may be the result of relatively recent processes, such as niche change or weak selection towards corn-base substrates. In contrast, for kefir-derived strains, many of the protein-coding genes, and even the pseudogene “archaeological records” of them, may be lost due to the long stay of these strains in a stable SCOBY consortium of kefir grains.

#### 3.3.2. Functional Annotation and Pan-Genomic Analysis

**Figure 7:**
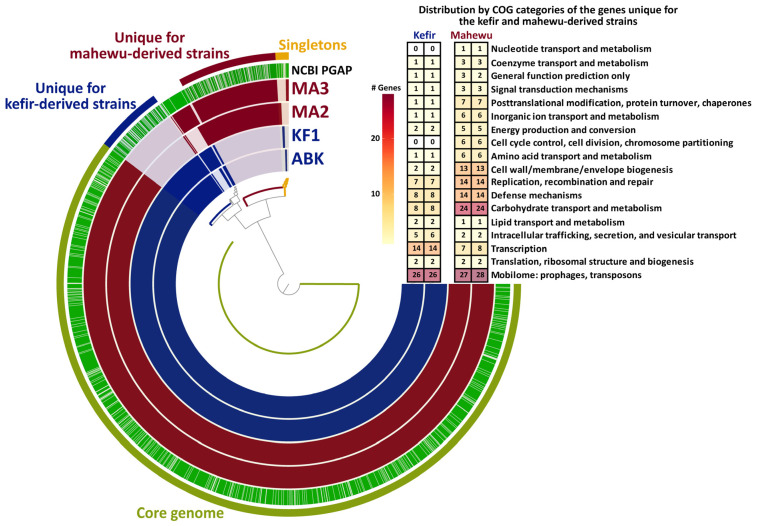
The Anvi’o diagram [45,46] representing the pan-genome analysis of kefir-derived (KF1 and ABK) and mahewu-derived (MA2 and MA3) strains of *L. paracasei*. Gene clusters (four inner rings) were ordered (inner dendrogram) according to their presence (solid color) or absence (grey color). The fifth ring depicts gene clusters that received functional annotation from the NCBI PGAP annotation pipeline [40], and the sixth ring depicts the binning of the pan-genome into core and accessory genomes. The later was further subdivided into gene clusters unique to either kefir- or mahewu-derived strains and strain-specific clusters (singletons). The heat map depicts the distribution by COG categories of the genes unique to either kefir- or mahewu-derived strains (“niche-specific genes”); niche-specific genes without an assigned COG category or assigned the “Unknown function” category were excluded from the heat map.

To reveal the specific genomic features of the studied *L. paracasei* strains that potentially could be associated with niche adaptations, functional annotations of the sequenced genomes and their comparative (i.e., pan-genomic) analysis were performed. For all sequenced genomes, approximately 83% of all predicted CDSs were functionally annotated by NCBI PGAP annotation pipeline [40], and approximately 76% were assigned to suitable clusters of orthologous groups of proteins (COG) by the Anvi’o [45,46] anvi-run-ncbi-cogs algorithm. Additionally, approximately 9% of all predicted CDSs were assigned to suitable KEGG pathway modules by the Anvi’o anvi-run-kegg-kofams algorithm, and the completeness of the modules (i.e., the completeness of the metabolic pathway encoded by the genes in these modules) was assessed by the Anvi’o anvi-estimate-metabolism algorithm. The results of the functional annotations are summarized in Supplementary Table S1. The comparison of the CDS content of the sequenced genomes and information about their main differences are shown in Figure 7.

Recently, to discuss the genomes of strains belonging to the same species, the concepts of pan- and core genome have been introduced [57]. While the pan-genome is the union of sets of genes from all considered genomes, the core genome is the intersection of these sets. All the genes from a pan-genome that do not belong to a core genome form an accessory genome. The genes from pan-, core and accessory genomes are grouped into clusters of homologous genes for further investigations [45,46].

For four sequenced *L. paracasei* strains, a total of 2592 gene clusters were identified in the pan-genome (Figure 7), and the core genome consisted of 2086 gene clusters. While 1975 gene clusters from the core genome contained only genes that presented in a single copy in each genome (i.e., single-copy orthologs), 111 clusters contained genes that presented in multiple copies in at least one of the studied genomes (i.e., paralogs). Importantly, all gene clusters from the complete KEGG pathway modules belonged to the core genome. This suggests that any strain-specific differences in the functional properties described in Section 3.2.3 and Section 3.2.4 are most probably the result of differing gene regulation, enzyme activities or both.

Importantly, the major part of the accessory genome of the studied *L. paracasei* strains consisted of gene clusters specific to either kefir- or mahewu-derived strains (Figure 7). Most of these “niche-specific genes” were related to the Mobilome COG category, which is closely related to genome stability. Although limited information regarding the genome stability of different *Lactobacillus* spp. is currently available, there are several pieces of evidences suggesting that genome stability influences trait stability to some extent and, hence, can be linked with a niche adaptation [58,59,60]. Furthermore, many niche-specific genes in *L. paracasei* genomes were related to transcriptional regulation, which again suggests the regulatory nature of strain-specific differences in the functional properties described in Section 3.2.3 and Section 3.2.4. Additionally, the multitude of niche-specific genes related to carbohydrate transport in the genomes of the mahewu-derived strains suggests the presence of regulation not only at the transcriptional level but at the level of the carbohydrate fluxes incoming into cells.

#### 3.3.3. Genome Stability

As most of the niche-specific gene clusters belonged to the Mobilome COG category, the genome stability of the *L. paracasei* strains was compared. The information about the main markers of genome stability, the presence of mobile genetic elements, prophages and plasmids [61], in the studied genomes of *L. paracasei* strains is shown in Table 3.

In terms of mobile genetic elements, the genomes of the kefir-derived strains carried on average 50 insertion sequences (ISs), and 64 such sequences on average were annotated in the genomes of the mahewu-derived strains. Genome analysis with ISFinder showed that, in addition to the IS families IS3, IS5, IS30, IS256 and ISL3 found in the genomes of the kefir-derived strains, the genomes of both mahewu-derived strains contained ISs from the IS6 family, and the *L. paracasei* MA2 genome contained ISs from the IS1182 family. While the majority of ISs detected in the genomes of the kefir-derived strains originated from *Lactobacillus* spp. (mainly *Lactobacillus rhamnosus* and *Lactobacillus casei*), the ISs detected in the genomes of the mahewu-derived strains mainly originated from *Lactococcus lactis*. It should be noted that *L. lactis* is a common strain found in spontaneously fermented mahewu [62,63,64,65,66,67]. Considering that the starter for the mahewu used in this study most likely originated from some traditionally prepared mahewu, the presence of many ISs from *L. lactis* in the genomes of the mahewu-derived strains can be explained by the close interactions of these LAB species in the original spontaneous fermentation. Although the exact role of ISs in the evolution of bacterial genomes is still debated, their general impact on the architecture of microbial genomes is undeniable [68]. It can be hypothesized that, due to the higher number of ISs, the genomes of the mahewu-derived strains have higher genome instability (plasticity) than those of the kefir-derived strains.

The search for prophage-containing regions showed that the genomes of all studied strains had only two incomplete prophage regions in common. The genomes of the kefir-derived strains exclusively contained two regions with incomplete prophages, one region with a questionable prophage and one region with an intact prophage. The genomes of the mahewu-derived strains exclusively contained one region with incomplete prophages and two regions with intact prophages. It should be emphasized that none of the studied strains contained CRISPR arrays in their genomes and, consequently, they were equally vulnerable to the incorporation of prophages. However, based on the lower number of intact prophages and the larger number of prophages inactivated by the accumulated mutations, it can be proposed that the kefir-derived strains encountered a lower number of recent prophage-incorporation events compared to the mahewu-derived strains.

In terms of extrachromosomal DNA, no plasmids were detected in the genomes of the kefir-derived strains, while the genomes of the mahewu-derived strains contained one 35.5 kbp plasmid. This plasmid encoded 39 genes, most of which were annotated as unknown. According to the BLAST search, the closest plasmid was plasmid pLDW-11 from *Companilactobacillus alimentarius* DSM 20249, with 96% sequence identity and 83% query coverage. Although the typical habitat (if there is one) of *C. alimentarius* is unknown [23], some of its strains were previously isolated from sourdough [69,70]. It can be proposed that the horizontal transfer of the plasmid from *C. alimentarius* to the mahewu-derived strains of *L. paracasei* occurred due to their interaction during the fermentation of corn inoculated by wheat flour, which is a typical process in mahewu fermentation [64].

#### 3.3.4. Bacteriocin Genome Content

There are several ways by which *Lactobacillus* spp. can inhibit growth of pathogenic microorganisms and each other. While the inhibiting properties of organic acids and peroxide produced by *Lactobacillus* spp. in the process of fermentation have been known for decades [71,72,73], production of specific antimicrobial proteins—bacteriocins—by these microorganisms is a relatively new discovery [74,75]. Bacteriocins are ribosomal synthesized peptides with antimicrobial activity [76,77]. It is currently believed that Gram-positive bacteria—in particular, LAB—produce bacteriocins with a broader spectrum of antimicrobial activity than Gram-negative bacteria, which produce bacteriocins that inhibit only a number of specific microorganisms typically encountered in their habitat [78,79,80].

Genome analysis with BAGEL4 showed that all the studied strains of *L. paracasei* contained in their genomes the following identical bacteriocin clusters (Table 4): Butyrivibriocin AR10, ComC/Lactococcin/LSEI_2386, Carnocin CP52 and LSEI 2163. Additionally, the genomes of the kefir-derived strains exclusively contained the Enterolysin A bacteriocin cluster, while the genomes of the mahewu-derived strains exclusively contained the ComC/Acidocin_8912/Acidocin A bacteriocin cluster. Hence, in total, both the kefir-derived strains and the mahewu-derived strains possessed five bacteriocin clusters in their genomes.

Interestingly, in the work of Ghosh et al. [81], who analyzed 75 strains of *L. paracasei*, the genomes of all studied strains contained two to three bacteriocin clusters on average, and the highest number of such clusters (five) was detected only in four strains. Almost all bacteriocin clusters detected in our strains were present in at least 40% of all the genomes studied by Ghosh et al. [81]. The exceptions were the Butyrivibriocin AR10 cluster, which was detected in the genomes of both kefir- and mahewu-derived strains, and the Acidocin 8912 cluster, which was detected only in the genome of the mahewu-derived strains. In the work of Ghosh et al. [81], the Butyrivibriocin AR10 cluster was present in the genomes of 5 out of 75 strains and Acidocin 8912 in the genomes of 4 out of 75 strains. Thus, a distinctive feature of the genomes of both kefir-derived and mahewu-derived strains was the presence of the Butyrivibriocin AR10 cluster, which is rarely observed in other *L. paracasei* strains. The additional peculiarity of the mahewu-derived strains was the absence in their genomes of the Enterolysin A bacteriocin cluster, which is relatively widespread among *L. paracasei* [81].

## 4. Conclusions

In this work, microbiological, biochemical and genomic analyses were utilized for comparative characterization of *L. paracasei* strains isolated from such non-standard environments as SCOBY—kefir grains (*L. paracasei* strains KF1 and ABK)—and the traditional corn-based nonalcoholic beverage of South Africa mahewu (*L. paracasei* strains MA2 and MA3). It was demonstrated that the biochemical and fermentation characteristics of the strains correlated with their isolation source. Moreover, the genomic analysis demonstrated that both kefir- and mahewu-derived strains possessed a number of gene clusters specific to strains of the same origin. The majority of these niche-specific gene clusters belonged to the Mobilome, Transcription and Carbohydrate Transport and Metabolism COG categories. It was also shown that the mahewu-derived strains possessed more flexible genome content (i.e., more pseudogenes, insertion sequences, intact prophages and plasmids) than the kefir-derived strains. It was proposed that the relative stability of the genomes of the kefir-derived strains reflects their long-term adaptation to the SCOBY environment.

From the technological perspective, all the studied strains demonstrated the ability to produce functional fermented products with antioxidant and antihypertensive properties, and the kefir-derived strains showed promising properties for their use in the recently proposed long-term fermentation processes (which has been proposed to be able to increase their resistance to gastrointestinal digestion and their intestinal adhesion ability). Additionally, all studied strains demonstrated the ability to inhibit growth of common pathogenic bacteria, which highlights their probiotic potential. Further studies of *L. paracasei* strains isolated from non-standard environments and their characterization at several levels, including metabolomic and proteomic, will not only provide a more complete picture of the transitional (nomadic) lifestyle of this LAB species but also help in discovering new strains that have potential health-promoting properties.

## Figures and Tables

**Table 1:** The antagonistic interactions between the studied strains of *L. paracasei*.

	Strains of *L. paracasei*		
	KF1	ABK	MA2
**ABK**	**-**		
**MA2**	**+**	**+**	
**MA3**	**+**	**+**	**-**

**Table 2:** Data on the genome sequencing of the kefir-derived (KF1 and ABK) and mahewu-derived (MA2 and MA3) strains of *L. paracasei*.

**Kefir-Derived Strains**							
***Lacticaseibacillus paracasei* KF1 (GB Accession: GCA_023470645.1)**				***Lacticaseibacillus paracasei* ABK (GB Accession: GCA_018967025.1)**			
**Sequencing**				**Sequencing**			
Sequencing technology	Ion Torrent	Number of reads	3,432,445	Sequencing technology	Ion Torrent	Number of reads	3,635,019
		Mean read size	208 bp			Mean read size	208 bp
**Assembly**		**Structural annotation**		**Assembly**		**Structural annotation**	
Assembly size, bp	2,697,398	Genes (total):	2791	Assembly size, Mb	2,698,106	Genes (total):	2796
Overall coverage	100×	- Protein coding	2517	Overall coverage	100×	- Protein coding	2524
Number of contigs	248	- RNA coding	78	Number of contigs	246	- RNA coding	78
Longest contig, bp	212,858	- Pseudogenes	196	Longest contig, bp	212,860	- Pseudogenes	194
N50 contig size, bp	36,612	CRISPR arrays	0	N50 contig size, bp	36,610	CRISPR arrays	0
Mean contig size, bp	10,572			Mean contig size, bp	10,773		
**Mahewu-Derived Strains**							
***Lacticaseibacillus paracasei* MA2 (GB Accession: GCA_018966985.1)**				***Lacticaseibacillus paracasei* MA3 (GB Accession: GCA_023470655.1)**			
**Sequencing**				**Sequencing**			
Sequencing technology	Ion Torrent	Number of reads	2,972,024	Sequencing technology	Ion Torrent	Number of reads	3,314,216
		Mean read size, bp	209 bp			Mean read size, bp	212 bp
**Assembly**		**Structural annotation**		**Assembly**		**Structural annotation**	
Assembly size, bp	2,878,977	Genes (total):	2977	Assembly size, Mb	2,870,266	Genes (total):	2965
Overall coverage	100×	- Protein coding	2651	Overall coverage	100×	- Protein coding	2650
Number of contigs	363	- RNA coding	79	Number of contigs	350	- RNA coding	79
Longest contig, bp	170,621	- Pseudogenes	247	Longest contig, bp	170,654	- Pseudogenes	236
N50 contig size, bp	37,017	CRISPR arrays	0	N50 contig size, bp	37,018	CRISPR arrays	0
Mean contig size, bp	7622			Mean contig size, bp	8022		

**Table 3:** Data on the genome stability of the kefir-derived (KF1 and ABK) and mahewu-derived (MA2 and MA3) strains of *L. paracasei*.

		Strains of *L. paracasei*			
		KF1	ABK	MA2	MA3
**Insertion sequences**					
**IS Family**	**Origin**	**BLAST hit**			
IS5	*Lactobacillus rhamnosus*	ISLrh2	ISLrh2	ISLrh2	ISLrh2
IS5	*Lactobacillus rhamnosus*	ISLrh3	ISLrh3	ISLrh3	ISLrh3
IS5	*Lactobacillus casei*	ISLca2	ISLca2	ISLca2	ISLca2
IS3	*Lactobacillus casei*	ISL1	ISL1	ISL1	ISL1
IS30	*Lactobacillus plantarum*	ISLpl1	ISLpl1	ISLpl1	ISLpl1
IS3	*Lactobacillus sanfranciscensis*	IS153	IS153	IS153	IS153
ISL3	*Leuconostoc mesenteroides*	IS1165	IS1165	IS1165	IS1165
IS30	*Pediococcus pentosaceus*	ISPp1	ISPp1	ISPp1	ISPp1
IS256	*Enterococcus hirae*	IS1310	IS1310	IS1310	IS1310
IS6	*Leuconostoc mesenteroides*	-	-	IS1297	IS1297
IS6	*Lactococcus lactis*	-	-	ISS1N	ISS1N
IS6	*Lactococcus lactis*	-	-	ISS1E	ISS1E
IS6	*Lactococcus lactis*	-	-	ISS1M	ISS1M
IS6	*Lactococcus lactis*	-	-	ISS1D	ISS1D
IS6	*Lactococcus lactis*	-	-	ISS1CH	ISS1CH
IS6	*Lactococcus lactis*	-	-	ISS1A	ISS1A
IS6	*Lactococcus lactis*	-	-	IS946V	IS946V
IS6	*Lactococcus lactis*	-	-	ISS1T	ISS1T
IS6	*Lactococcus lactis*	-	-	ISS1S	ISS1S
IS6	*Lactococcus lactis*	-	-	ISS1RS	ISS1RS
IS6	*Lactococcus lactis*	-	-	ISS1B	ISS1B
IS6	*Lactococcus lactis*	-	-	ISS1X	ISS1X
IS6	*Lactococcus lactis*	-	-	ISS1Z	ISS1Z
IS6	*Lactococcus garvieae*	-	-	ISLgar4	ISLgar4
IS5	*Streptococcus thermophilus*	-	-	IS1194	-
IS1182	*Streptococcus agalactiae*	-	-	ISSag8	-
IS1182	*Streptococcus agalactiae*	-	-	IS1563	-
**Prophages**					
**Most common phage name**	**Completeness**	**Number of Total Proteins**			
PHAGE_Lactob_phijl1_NC_006936	Intact	57	59	-	-
PHAGE_Lactob_BH1_NC_048737	Questionable	29	28	-	-
PHAGE_Lactob_iLp84_NC_028783	Incomplete	18	18	-	-
PHAGE_Staphy_phiPV83_NC_002486	Incomplete	9	10	-	-
PHAGE_Staphy_SPbeta_like_NC_029119	Incomplete	22	19	23	19
PHAGE_Lactob_iLp1308_NC_028911	Incomplete	29	26	29	29
PHAGE_Lister_LP_101_NC_024387	Intact	-	-	19	19
PHAGE_Lactob_iA2_NC_028830	Intact	-	-	48	48
PHAGE_Lactob_Lc_Nu_NC_007501	Incomplete	-	-	16	16
**Plasmids**					
**Best BLAST hit**	**Origin**	**Presence**			
pLDW-11	*Companilactobacillus alimentarius* DSM 20249	No	No	Yes	Yes

**Table 4:** Data on the bacteriocin genome content of the kefir-derived (KF1 and ABK) and mahewu-derived (MA2 and MA3) strains of *L. paracasei*.

Bacteriocin-Containing Cluster	Strains of *L. paracasei*			
	KF1	ABK	MA2	MA3
Butyrivibriocin AR10	Yes	Yes	Yes	Yes
ComC/Lactococcin/LSEI_2386	Yes	Yes	Yes	Yes
Carnocin CP52	Yes	Yes	Yes	Yes
LSEI 2163	Yes	Yes	Yes	Yes
ComC/Acidocin 8912/Acidocin A	No	No	Yes	Yes
Enterolysin A	Yes	Yes	No	No

## Data Availability

Data is contained within the article or supplementary material.

## Supplementary Materials

The following supporting information can be downloaded at: https://www.mdpi.com/article/10.3390/foods12010223/s1, Table S1: Summary of the genome annotations.
